# PERMA-Profiler for adolescents: validity evidence based on internal structure and related constructs

**DOI:** 10.3389/fpsyg.2024.1415084

**Published:** 2024-07-12

**Authors:** Iorhana Fernandes, Daniela Sacramento Zanini, Evandro Morais Peixoto

**Affiliations:** ^1^Department of Psychology, Pontifícia Universidade Católica de Goiás, Goiânia, Brazil; ^2^Department of Psychology, Universidade São Francisco, Campinas, Brazil

**Keywords:** PERMA-Profiler, well-being, adolescence, psychometry, psychological assessment

## Abstract

**Introduction:**

The PERMA model of well-being has gained prominence in the study of well-being by the Positive Psychology movement. However, the model has been little studied regarding its applicability in different populations, such as adolescents. This study sought to evaluate the psychometric properties of the PERMA-Profiler instrument for Brazilian adolescents, as well as the measurement invariance for different age groups and gender, and investigate the relation with external variables.

**Methods:**

Confirmatory Factor Analysis and Multigroup Confirmatory Factor Analysis were used to test the internal structure and invariance of the PERMA-Profiler. Reliability was determined with McDonald’s Omega and composite reliability. A total of 1,197 adolescents between 11 and 19 years old from different regions of Brazil participated.

**Results:**

The results of the confirmatory factor analysis indicated that the five correlated factors model was the most appropriate, presenting good factor loadings and adequate reliability. The scale proved to be invariant for adolescents of different age groups and gender. Correlations with associated variables were significant and moderate to strong, showing positive relations between positive emotions and well-being, and negative relations with negative affects and depressive and anxiety symptoms.

**Discussion:**

These results contribute to the understanding of well-being in adolescence and highlight the importance of promoting different components of well-being for adolescents’ mental health.

## Introduction

The PERMA model of well-being, an acronym for Positive Emotions (P), Engagement (E), Positive Relationships (R), Meaning (M), and Achievements (A), was developed by Seligman (2011) with the aim of creating a theoretical model that addresses the central aspects of well-being and the integration of eudaimonic and hedonic conceptions. This model has spread quickly around the world (Bazargan-Hejazi et al., 2021) and has been applied in different contexts with adult populations (e.g., O’Connor et al., 2016; Tansey et al., 2017; Turner and Thielking, 2019; Umucu et al., 2020; Blaire and Simmons, 2021; Tu et al., 2021). To evaluate the model, Butler and Kern (2016) developed the PERMA-Profiler instrument, which has been adopted by different researchers and translated into several languages (e.g., Cobo-Rendón et al., 2020; Giangrasso, 2021; Zewude and Hercz, 2022; Alves et al., 2023; Chaves et al., 2023; Mahamid et al., 2023).

The PERMA-Profiler scale is composed of 15 items that assess the PERMA components and eight additional items that evaluate happiness, negative emotions (sadness, anger, and anxiety), loneliness and physical health. The final measure consists of 23 items, scored on a Likert scale ranging from 0 = never to 10 = always. Regarding psychometric indicators, the authors found a factor structure of five correlated factors and three items in each factor, with good fit indices and good reliability indicators. These factors are: positive emotions (*α* = 0.88), engagement (*α* = 0.72), relationships (*α* = 0.82), meaning (*α* = 0.90), and achievements (*α* = 0.79).

Although the original instrument presents good psychometric indicators and a factorial structure of five correlated factors, in subsequent adaptations some authors have found divergences regarding the internal structure of the model. Bartholomaeus et al. (2020) tested different models: unidimensional, multifactorial (five correlated factors), and hierarchical, which corresponds to the five PERMA components, comprising a general well-being factor. The unidimensional model was not accepted. The five correlated factors model and the second-order model had marginally different fit indices. The authors indicated that the second-order factorial structure might be the most appropriate to understand PERMA, due to the theoretical understanding of some authors (Goodman et al., 2017; Sellbom and Tellegen, 2019) that PERMA represents several facets of a single construct, which is well-being. However, predominantly, studies have pointed to a structure of five factors correlated with each other (Cobo-Rendón et al., 2020; Giangrasso, 2021; Wammerl et al., 2019; Zewude and Hercz, 2022; Alves et al., 2023; Chaves et al., 2023; Mahamid et al., 2023).

Such evidence corresponds to adaptations of the instrument for healthy adults, highlighting a gap in studies on different populations according to age group and gender. To date, only two studies have been found with adolescent populations using the PERMA-Profiler scale: one with Argentine adolescents (Waigel and Lemos, 2023) and the other with Indian adolescents (Singh and Raina, 2020). In both studies, the factor structure of five correlated factors had the best fit to the data, consistent with the original instrument. In Brazil, no adaptations have yet been found for this population.

Although there are few studies with the PERMA-Profiler instrument, evidence of the viability of the PERMA model applied to adolescence can be found in the literature. With regard to positive emotions, such as joy, contentment, and pleasure, Wu et al. (2022) point out that adolescents with high scores in positive emotions and low scores in negative emotions tend to have fewer mental health problems, such as depressive and anxiety symptoms, and better levels of self-esteem, gratitude, resilience, and life satisfaction.

Engagement is related to ‘losing track of time’ while carrying out a certain activity (Usán and Salavera, 2019). Adolescents with a higher level of engagement tend to have lower consumption of substances such as tobacco and alcohol (Pérez-Fuentes et al., 2021), a positive attitude toward authorities and higher self-esteem (Zhao et al., 2021). It is noteworthy that this component has a comprehensive nature and can be evaluated in different contexts, such as leisure activities with friends or family, activities carried out in the community, among others. Some authors have pointed out that engagement in leisure activities that involve peers predicts well-being in terms of levels of positive emotions and mental health (Asquith et al., 2022), as does engagement in sports and vacation trips or holidays enhance the perception of life satisfaction (Schmiedeberg and Schröder, 2017).

Regarding positive relationships, Carrillo et al. (2021) investigated, through a literature review, children and adolescents’ understanding of well-being. Among the most reported aspects are healthy and supportive relationships, which relate to receiving adequate treatment from others and maintaining bonds with other people (adults and peers). Children and adolescents understand that relationships in which they are treated well promote well-being, as well as protection, security, experience of positive emotions and support for daily life (Carvalho et al., 2021).

Meaning concerns the significance attributed to one’s existence and the feeling of belonging and connection with something or someone believed to be greater than oneself, for example God, church, family, etc. (Krok, 2018). This construct is defined as the sense that life has meaning, direction and intentionality, and that this sense plays a guiding role in establishing life goals and making decisions regarding the use of personal resources (Ribeiro et al., 2020). In adolescents, evidence points to meaning as a moderating protective factor in the relation to suicidal ideation (Zhang et al., 2017; Simões et al., 2022), and also as a factor against involvement in violent behavior or being a victim of peer violence (Zawadzka et al., 2018).

Achievement is about making progress toward a goal and being recognized internally and externally for it (Seligman, 2011). Studies show that high levels of recognition of one’s own competence and achievements predict high levels of psychological well-being (Krok, 2018).

### The present study

To fill this gap, this study aims to estimate the psychometric properties of the PERMA-Profiler scale for Brazilian adolescents. Specifically, the following aspects will be evaluated: (I) the internal structure of the scale, contrasting four factorial models suggested by the literature, namely, one factor, hierarchical, five correlated factors and bifactor models; (II) the scale’s reliability indicators; (III) the measurement invariance in relation to gender and age group; and (IV) the correlation of PERMA with related variables.

## Methods

### Participants

One thousand, one hundred ninety-seven adolescents aged between 11 and 19 years old participated in this study (*M* = 15.3, *SD* = 1.65), who identified themselves as cisgender girls (54.3%), cisgender boys (41.6%), non-binary (3%) and transgender (1.1%). These participants were from all five of Brazil’s regions: Central-West (42.5%), Southeast (52.8%), South (3.1%), Northeast (1.1%), and North (0.9%). Adolescents answered the questionnaires via cell phone (55.6%), computer (14.5%) and tablet (29.8%).

To participate in this research, participants must be between 10 and 19 years old. Their guardians must agree to participate by signing the Free and Informed Consent Form, and the adolescents themselves must agree to participate through the Term of Free and Informed Assent. Participants who self-report any neurological impairment that impedes their ability to read and understand the items presented will be excluded.

### Instruments

#### Sociodemographic questionnaire

A sociodemographic questionnaire was developed containing questions regarding age, gender identification, education, housing and income.

#### PERMA-Profiler for adolescents

The original scale (Butler and Kern, 2016) is composed of 23 self-report items scored from 0 to 10. The measure is composed of 15 core items that assess the PERMA components and eight additional items that evaluate positive emotions, loneliness, happiness, and perception of health. The factor structure of five correlated factors showed good indicators [*χ*^2^(80) = 10.61; CFI = 0.97; TLI = 0.96 RMSEA = 0.06; SRMR = 0.03], as well as satisfactory internal consistency (Positive emotions *α* = 0.88; Engagement *α* = 0.72; Relationships *α* = 0.82; Meaning *α* = 0.90; Achievements *α* = 0.79). To calculate the score for the five PERMA components, the arithmetic mean of the three items in each domain is taken. Negative emotions and health perception are calculated by the arithmetic mean of the three items, for example: (P1 + P2 + P3)/3 = Positive emotions component score. Happiness and loneliness are assessed by a single item.

#### Kessler K10

To evaluate psychological distress, the Kessler Psychological Distress Scale (K10) was used, adapted into Portuguese by Peixoto et al. (2021) for individuals aged 14 and over. The instrument consists of 10 self-report items that assess emotional stress in the last 30 days, scored on a 5-point Likert scale, with 1 = never and 5 = all the time. To interpret the scale, all items must be added together, and higher scores mean higher levels of anguish or psychological suffering. Due to the factorial structure of the instrument, it is also possible to know the levels of anxiety and depression separately. Anxiety is the sum of items 2, 3, 5, and 6. Depression is the sum of items 1, 4, 7, 8, 9, and 10. The instrument in original study has shown good psychometric indices and desirable reliability (*α* = 0.87).

#### Life satisfaction

The Global Life Satisfaction Scale for Adolescents (EGSV-A) was developed by Giacomoni et al. (2012), and it is a Likert scale composed of 10 statements that are scored from 1 (not at all) to 5 (very much). The level of life satisfaction is known through the sum of all items answered. Regarding internal consistency, the scale presented good reliability indexes (*α* = 0.90).

#### Positive and negative affects

The Positive and Negative Affects Scale for Adolescents (EAPN-A) was validated for Brazilian adolescents by Segabinazi et al. (2012), and it is a five-point Likert scale consisting of 28 items referring to positive and negative affects. Adolescents must mark how much they feel that way, ranging from 1 – “not at all” to 5 – “very much,” and its application can be individual or collective. To know the result, the raw score must be calculated, which is obtained by adding the answered items for each construct (PA and NA). Scale validation studies demonstrate that the internal consistency of the scale assessed using Cronbach’s alpha was 0.88 for PA and NA.

#### Cross-cultural adaptation procedure

With regard to the cross-cultural adaptation of the instrument and content validity, the International Test Commission (International Test Commission, 2017) translation and adaptation test checklist was used, along with the recommendations suggested by Borsa et al. (2012). The adaptation process comprised five stages: (I) Translation of the instrument; (II) Synthesis of translations; (III) Assessment by expert judges; (IV) Summary of evaluations; and (V) Evaluation by target audience. The first stage involved translation by two independent translators: a specialist in the subject of well-being and psychometrics in English, and a translator specialized in English. After the translations, a synthesis was carried out, considering semantic equivalence, idiomatic equivalence, experiential equivalence and conceptual equivalence, as proposed by Borsa et al. (2012). Based on the synthesis of translations, the language of the items was adapted for the adolescent audience (between 11 and 19 years old), and submitted to four independent judges who are doctors and experts in psychometrics, on the subject of well-being and adolescence. These judges evaluated the scale for clarity, pertinence, and relevance. After this stage, an analysis of the judges’ contributions was carried out, and their suggestions were incorporated into the scale. The revised version of the instrument was then administered to 30 adolescents, who assessed the clarity and understanding of the items through structured interviews (Fernandes and Zanini, manuscript under construction). Based on the adolescents’ feedback, the final version of the instrument was defined.

#### Data collection procedure

Participants were contacted in two ways. The first, through organic dissemination through the social media channels of the researchers involved, with the aim of reaching adolescents in different environments. In the second format, adolescents were contacted through partner schools in different regions of the country and, after consent from their guardians, the questionnaires were administered at the educational institution via computer or cell phone.

#### Ethical procedures

This research was approved by the Brazilian research ethics committee under number CAAE: 53061221.9.0000.0037. All participants and their guardians, respectively, signed the Free and Informed Assent Form and the Free and Informed Consent Form.

### Data analysis

To evaluate the factorial structure of the scale, a Confirmatory Factor Analysis (CFA) was conducted using the Mplus software (Muthén and Muthén, 2012), with the Weighted Least Square estimation method (WLSMV). Four models were tested, namely: (I) One general factor model, in which all items compose a general well-being factor (Ryan et al., 2019); (II) Hierarchical model, which consists of five PERMA factors at the first level and one general factor at the second level (Bartholomaeus et al., 2020); (III) Five correlated factors model, in which each PERMA factor is composed of three specific items, and all factors are correlated (Butler and Kern, 2016); and (IV) Bifactor model, which corresponds to all items loading on a general well-being factor and the items loading on each PERMA component (Wammerl et al., 2019). Unlike the hierarchical model, the bifactor model assumes that the associations of the general well-being factor with the responses to the observed items are direct and independent of the associations of the PERMA factors with the responses to the items. For all models, only the 15 central items of the instrument were used.

To interpret the results, the following fit indices were adopted: *χ*^2^ (chi-square), df (degrees of freedom), RMSEA (Root-Mean-Square Error of Approximation), CFI (Comparative Fit Index), and TLI (Tucker–Lewis Index). The following values were used as parameters: CFI and TLI ≥ 0.90, RMSEA values ≤0.08 and *χ*^2^/df ≤ 5 (Muthén and Muthén, 2017; Tabachnick and Fidell, 2019).

A multigroup confirmatory factor analysis (MGCFA) was performed to investigate the invariance of the PERMA-Profiler for adolescents according to gender and age group (11–15 years and 16–19 years). MGCFA evaluated measurement invariance in three models, namely: configural, metric and scalar. Model 1 (configural invariance) assessed whether the scale configuration (number of factors and items per factor) was acceptable for both groups (male and female). If the model is not supported, the factorial structure of the instrument cannot be considered equivalent for the groups evaluated. Model 2 (metric invariance) analyzed whether the factor loadings of the items could be considered equivalent between the groups. Model 3 (scalar invariance) investigated whether the level of latent trait required to endorse item categories (thresholds) was equivalent across groups (Cheung and Rensvold, 2002). Measurement invariance was assessed using the CFI difference test (ΔCFI, Cheung and Rensvold, 2002). If, when setting a parameter, a significant reduction in CFI indices is found (ΔCFI >0.01), measurement invariance cannot be accepted (Cheung and Rensvold, 2002).

To evaluate the internal consistency of the instrument, McDonald’s omega was estimated, obtained from the Jamovi software (Version 2.3; The Jamovi Project, 2022), and composite reliability using the Composite Reliability Calculator (Colwell, 2016). To interpret the coefficients, values ≥0.70 were adopted as good indicators of precision (Tabachnick and Fidell, 2019). To evaluate the evidence based on the relation with external variables, Pearson correlation analyzes were carried out (*r*). To interpret the results, correlations with *r* between −0.09 and 0.09 are interpreted as null, *r* between 0.10 and 0.29 small, *r* between 0.30 and 0.49 medium or moderate, and *r* between 0.50 and 1.0 strong (Cohen, 1988). For this purpose, the Jamovi software was used (The Jamovi Project, 2022).

## Results

Table 1 presents the fit indices of the four models tested in this study. The one factor model presented unacceptable fit indices. The bifactor and hierarchical models presented reasonable indices, although the RMSEA was higher than expected. The five correlated factors model presented the best fit to the data, with good indicators and adequate RMSEA, especially when considering the confidence interval. It is also noteworthy that models with few factors and items are expected to consequently produce a higher RMSEA (Kenny et al., 2015).

**Table 1:** Confirmatory factor analysis (CFA) results for the four contrasted models.

Model	*χ* ^2^	df	*χ*^2^/df	CFI	TLI	RMSEA (90% CI)
One factor	2410.105	90	26.778	0.894	0.877	0.163 (0.158–0.169)
Hierarchical	1495.714	85	17.596	0.936	0.921	0.131 (0.125–0.137)
Five factors	700.287	80	8.753	0.972	0.963	0.090 (0.084–0.096)
Bifactor	1467.433	75	18.342	0.937	0.911	0.139 (0.132–0.145)

The factor loadings, as well as the reliability indicators, are presented in Table 2. The items have good factor loadings (0.319–0.884) and good reliability indicators with omega ranging from 0.77 to 0.89 and composite reliability from 0.79 to 0.90. The MGCFA results are presented in Table 3 considering gender and age group (adolescents between 11 and 15 years old and 16 and 19 years old). The results support configural, metric and scalar invariance, demonstrating that the PERMA-Profiler instrument for adolescents is an equivalent measure for men and women, and for younger (11–15 years old) and older (16–19 years old) adolescents, which allows comparison between groups.

**Table 2:** Factor loadings of the five correlated factors model and reliability indicators.

Items	P	E	R	M	A
Item 3	0.831				
Item 13	0.848				
Item 22	0.884				
Item 2		0.685			
Item10		0.779			
Item17		0.319			
Item 8			0.696		
Item 19			0.825		
Item 21			0.729		
Item 7				0.831	
Item 9				0.871	
Item 20				0.883	
Item 1					0.742
Item 5					0.849
Item 15					0.639
McDonald’s *ω*	0.877	0.635	0.776	0.886	0.770
Composite reliability	0.890	0.636	0.795	0.897	0.790

**Table 3:** Multigroup confirmatory factor analysis (MGCFA) for men, women and age group.

Gender	RMSEA (90% CI)	CFI	TLI	ΔCFI
Configural invariance	0.092 (0.086–0.098)	0.969	0.960	–
Metric invariance	0.085 (0.079–0.091)	0.972	0.966	+0.003
Scalar invariance	0.057 (0.052–0.062)	0.979	0.984	+0.007

Age	RMSEA (90% CI)	CFI	TLI	ΔCFI
Configural invariance	0.092 (0.085–0.098)	0.971	0.962	-
Metric invariance	0.086 (0.080–0.092)	0.973	0.966	+0.002
Scalar invariance	0.059 (0.054–0.064)	0.978	0.984	+0.005

The Table 4 presents the correlations with external variables. The PERMA scale showed a significant correlation with all instruments (*p* < 0.001). PERMA components correlate with each other, with moderate to strong magnitude. The engagement and achievement components present a moderate magnitude correlation with life satisfaction (*r* = 0.39; 0.42) and positive affects (*r* = 0.48). Likewise, relationships present a moderate magnitude correlation between meaning (*r* = 0.65) and positive affects (*r* = 0.49). The other PERMA components show a strong correlation with the indicators of life satisfaction and positive affects (*r* = 0.63–0.73).

**Table 4:** Correlations between PERMA, life satisfaction, positive and negative affects, and psychological distress.

	1	2	3	4	5	6	7	8	9	10
1-P	—									
2-E	0.535*	—								
3-R	0.709*	0.482*	—							
4-M	0.732*	0.599*	0.650*	—						
5-A	0.512*	0.683*	0.475*	0.637*	—					
6-SATIS	0.734*	0.390*	0.634*	0.676*	0.424*	—				
7-PA	0.657*	0.482*	0.498*	0.565*	0.481*	0.631*	—			
8-NA	−0.508*	−0.150*	−0.388*	−0.443*	−0.212*	−0.537*	−0.193*	—		
9-DIS	−0.570*	−0.194*	−0.427*	−0.495*	−0.244*	−0.598*	−0.332*	0.730*	—	
10-ANS	−0.397*	−0.119*	−0.300*	−0.360*	−0.177*	−0.426*	−0.202*	0.622*	0.874*	—
11-DEP	−0.598*	−0.222*	−0.447*	−0.510*	−0.256*	−0.631*	−0.375*	0.699*	0.944*	0.665*

*p < 0.001; P, positive emotions; E, engagement; R, relationships; M, meaning; A, achievements; SATIS, life satisfaction; PA, positive affects; NA, negative affects; DIS, distress; ANS, anxiety; DEP, depression.

Positive emotions correlated with negative affects (*r* = 0.50), distress (*r* = 0.57) and depression (*r* = 0.59), with a strong magnitude, just as the meaning component correlated with depression (*r* = 0.51) with strong magnitude. Positive emotions correlated with anxiety (*r* = 0.50) with moderate magnitude. Engagement showed a low correlation with all risk factors (negative affects, distress, anxiety and depression [*r* = 0.15–0.22]). Also, meaning correlated with a moderate magnitude with negative affects (*r* = 0.44), distress (*r* = 0.49) and anxiety (*r* = 0.36). The achievement component presents a significant correlation with negative affects, distress, anxiety, and depression, however with low magnitude (*r* = 0.17–0.21).

## Discussion

The present study aimed to adapt and estimate the psychometric properties of the PERMA-Profiler scale for adolescents, in addition to contrasting four different models, evaluating the measurement invariance regarding age and gender, and the relation with external variables. In short, the scale presented good psychometric indicators and a factorial structure of five first-order correlated factors. The scale also proved to be suitable for assessing well-being in the PERMA model for adolescents of different age groups and in relation to gender.

The results of the confirmatory factor analysis indicate that the factor structure of five correlated factors is the most appropriate fit to understand the data. Similar results were found in the original version of the instrument (Butler and Kern, 2016) for Brazilian adults (Carvalho et al., 2021), for Indian and Argentine adolescents (Singh and Raina, 2020; Waigel and Lemos, 2023), and in several populations around the world (Cobo-Rendón et al., 2020; Giangrasso, 2021; Zewude and Hercz, 2022; Chaves et al., 2023; Mahamid et al., 2023). However, the factor structure of the PERMA model has been discussed.

Goodman et al. (2017) and Sellbom and Tellegen (2019) suggest that PERMA should be understood as a construct that is organized in a hierarchical structure, since the different facets compose well-being. This argument has been reinforced by slightly different fit indices when comparing the five first-order correlated factors model and the hierarchical model (Bartholomaeus et al., 2020). However, understanding the PERMA model applied to adolescence based on the factorial structure of five correlated factors corresponds to the essential properties indicated by Seligman (2018), which are: (I) Contribute to the construction of well-being; (II) People should seek it for itself, and not to promote another component; (III) It is defined and measured independently of another component.

Therefore, understanding PERMA from a first-order correlated model is close to the properties highlighted by Seligman (2018), and also to the instrument’s original proposal, since the authors suggest the possibility of well-being profiles that would be more adaptive for each context (Butler and Kern, 2016). In other words, depending on the context, presenting high levels of a PERMA component can be a risk factor, while in other contexts it can be beneficial (e.g., a high level of work engagement can be a risk factor for burnout in a work environment with high demands and few resources). In this way, it is possible to think of an intervention that focuses only on conscious engagement in certain actions, without interventions in the other PERMA components.

From this perspective, the first-order correlated model privileges the independence between variables and the possibility of interventions focused on promoting just one component. On the other hand, hierarchical models favor the promotion of the general factor, that is, intervening in one of the components will (theoretically) affect the general perception of well-being, since the second-order components have dependence between them, shared in the general factor. Thus, understanding the PERMA structure as a hierarchical model (as proposed by Goodman et al., 2017) can affect the objective of the interventions, which now have as its main objective the promotion of the general factor (well-being), and to a lesser extent proportion, the promotion of each component.

Regarding reliability, all factors presented good indicators. The engagement component presents the lowest indicator (*ω* = 0.63 CR = 0.63). This data corroborates what is found in national and international literature in studies adapting the PERMA-Profile instrument in different cultures with adolescents (Singh and Raina, 2020; Waigel and Lemos, 2023) and with adults (Wammerl et al., 2019; Carvalho et al., 2021; Alves et al., 2023). A possible explanation for this aspect is due to the specificity/amplitude of the engagement measure, since engagement behavior may refer to a specific context or activity (e.g., playing), and may not necessarily be generalizable to other contexts or activities (e.g., study).

Another factor that must be considered concerns item 17, which has the lowest factor loading (0.319), and corresponds to the flow construct indicator. Flow is understood as complete engagement, in which the individual loses track of the passage of time and there is full cognitive, emotional and psychological functioning (Csikszentmihalyi, 1998). It is noteworthy that measuring flow together with engagement and only through a single item can contribute to the lower factor loading in relation to the other items, and the consequent reliability indicator.

The need to include more items to assess engagement in adolescents is highlighted, as well as to elucidate the contexts that will be added in the assessment of the component. Although school engagement is traditionally adopted in adolescence (Krok, 2018), studies have emphasized the importance of engagement in leisure activities for building well-being (Carrillo et al., 2021). Furthermore, Fernandes and Zanini (manuscript under construction), points out the tendency of adolescents to interpret engagement as related to leisure activities with family and peers.

Regarding the assessment of scale invariance in relation to age and gender, configural, metric and scalar invariance analyzes were conducted. The results indicate measurement invariance, that is, the equivalence of the factorial structure both among younger adolescents (aged between 11 and 15 years old) and among older adolescents (aged between 16 and 19 years old), as well as among boys and girls. Similar results regarding invariance based on gender were observed in the study by Reinhardt et al. (2020), which examined the Mental Health Continuum-Short form instrument (Keyes, 2006) to assess mental well-being in adolescents. The importance of evaluating invariance for different gender identities is highlighted, and not just for gender. However, in this study, it was not feasible to carry out such an analysis, due to the small number of participants with diverse gender identities.

With regard to the relation between the PERMA components, we first point to the strong and moderate correlation between the components. Such evidence corroborates the PERMA proposal (Seligman, 2011, 2018), which theoretically proposes strong relations between the variables, however, maintaining their independence. As well as studies that show the correlation between the hedonic and eudaimonic dimensions of well-being (Kern et al., 2015; Krok, 2018; Cabrera et al., 2020; Mesurado et al., 2021). Furthermore, moderate to strong correlations were found between the PERMA components and subjective well-being in the dimensions of positive affects and life satisfaction, indicating the relation between positive aspects and the different models of well-being. Such results are repeated in several studies (Tansey et al., 2017; Lai et al., 2018; Choi, 2021; Donaldson et al., 2021).

In addition to the positive correlations between PERMA and positive variables, inverse correlations were found between PERMA and negative variables. PERMA was inversely related to negative affects, distress, depression and anxiety, with weak to moderate magnitude. Well-being in general is inversely related to negative health outcomes, such as anxiety and depression (Kern et al., 2015; Lai et al., 2018; Trigg, 2021). These associations between variables allow the interpretation that well-being variables may have a bidirectional correlation between positive and negative outcomes.

On the other hand, the engagement and achievement components present the weakest correlations with risk variables. Regarding engagement, the scope of the construct and the reliability indicators, already discussed in this study, must be considered. Such aspects can influence the correlations with the variables studied. Concerning achievement, one must consider the understanding of the construct in the adolescent population. In a study carried out by Fernandes and Zanini (manuscript under construction), the authors indicate that this component, for adolescents, is related to future goals, different from the understanding developed by adults, which is related to actions already completed (Seligman, 2011). Therefore, for the adolescent population, this component may present itself differently in relation to other variables.

In conclusion, this study offers in-depth insight into the adaptation and psychometric properties of the PERMA-Profiler scale for adolescents, highlighting the factor structure of five correlated factors as a robust model for understanding well-being in this specific group. This understanding aligns with the properties indicated by Seligman (2018), emphasizing the independent contribution of each component to the construction of well-being. This approach enables interventions focused on a specific component, recognizing the relevance of different well-being profiles in different contexts (Butler and Kern, 2016).

The results also point to the need to expand the evaluation of the engagement component, recognizing the diversity of contexts in which engagement is manifested in adolescence. While the measurement invariance was observed between different age groups and between sexes, the analysis also suggests the importance of expanding the investigation to include different gender identities. Furthermore, the correlations between the PERMA components and related variables reinforce the relevance of these elements in promoting well-being and reducing risk factors associated with mental health. However, the achievement component should be further studied in the adolescent population.

This study represents a significant advance in understanding well-being in adolescence, offering valuable insights into the structure and applicability of the PERMA model in a crucial developmental context. The findings from this study have significant practical implications, especially in the fields of education and mental health. The PERMA-Profiler scale for adolescents provide a robust tool for assessing well-being in this demographic. Educational policies can leverage this tool to monitor and enhance student well-being, implementing targeted interventions that address specific components of the PERMA model. In mental health practices, the validated PERMA-Profiler can aid in the early identification of adolescents at risk for negative outcomes such as anxiety and depression. Mental health professionals can develop tailored intervention programs that focus on enhancing particular aspects of well-being, such as positive relationships and meaning, to mitigate these risks.

However, some limitations of the research deserve to be highlighted. The limitations of this study can be indicated first in relation to the characteristics of the sample, which, although it represents part of the adolescent population in number, does not represent the diversity of gender and regions of the country. Therefore, it is suggested that future studies investigate the PERMA model in different populations in terms of sociodemographic and specific characteristics.

Furthermore, the cross-sectional research design used in this study to evaluate the correlation with related variables prevents the inference of causality between the variables, which limits their understanding. Self-reported data can be influenced by the respondent’s current mood, social desirability, and recall accuracy, potentially skewing the results. Future research should consider incorporating multiple data sources, such as teacher reports, peer assessments, and behavioral observations, to triangulate findings and provide a more comprehensive understanding of adolescent well-being. Additionally, longitudinal studies would help in understanding the stability of the PERMA components over time and their impact on long-term outcomes, as well as estimating the relation with external variables.

## Data availability statement

The datasets used and/or analyzed during the current study are available from the corresponding author on reasonable request. Requests to access the datasets should be directed to IF, iorhanafernandes@hotmail.com.

## Ethics statement

The studies involving humans were approved by Ethics Committee – Pontifícia Universidade Católica de Goiás – Approval number: 53061221.9.0000.0037. The studies were conducted in accordance with the local legislation and institutional requirements. Written informed consent for participation in this study was provided by the participants’ legal guardians/next of kin. Written informed consent was obtained from the minor(s)’ legal guardian/next of kin for the publication of any potentially identifiable images or data included in this article.

## Author contributions

IF: Writing – review & editing, Writing – original draft, Visualization, Validation, Software, Resources, Project administration, Methodology, Investigation, Formal analysis, Data curation, Conceptualization. DZ: Writing – original draft, Project administration, Formal analysis, Writing – review & editing, Visualization, Supervision, Methodology, Investigation, Conceptualization. EP: Validation, Writing – review & editing, Supervision, Software, Methodology, Formal analysis, Data curation.
